# Assessment of the quality of brain regions and neuroimaging metrics as biomarkers of Alzheimer’s Disease

**DOI:** 10.1186/2197-7364-2-S1-A46

**Published:** 2015-05-18

**Authors:** Tânia Faria Vaz, Filipa Lucena, Joana Pé-Leve, André Santos Ribeiro, Luís Lacerda, Nuno da Silva, David Nutt, John McGonigle, Hugo Alexandre Ferreira

**Affiliations:** Institute of Biophysics and Biomedical Engineering, Faculty of Sciences, University of Lisbon, Portugal; Lisbon School of Health Technology, Polytechnic Institute of Lisbon, Portugal; Centre for Neuropsychopharmacology, Division of Brain Sciences, Department of Medicine, Imperial College London, UK; Centre for Neuroimaging Sciences, Institute of Psychiatry, Kingâ€™s College London, UK; Institute of Neuroscience and Medicine 4, Forschungszentrum Jülich GmbH, Germany

Alzheimer Disease (AD) is characterized by progressive cognitive decline and dementia. Earlier diagnosis and classification of different stages of the disease are currently the main challenges and can be assessed by neuroimaging. With this work we aim to evaluate the quality of brain regions and neuroimaging metrics as biomarkers of AD. Multimodal Imaging Brain Connectivity Analysis (MIBCA) toolbox functionalities were used to study AD by T1weighted, Diffusion Tensor Imaging and 18FAV45 PET, with data obtained from the AD Neuroimaging Initiative database, specifically 12healthy controls (CTRL) and 33 patients with early mild cognitive impairment (EMCI), late MCI (LMCI) and AD (11 patients/group). The metrics evaluated were gray-matter volume (GMV), cortical thickness (CThk), mean diffusivity (MD), fractional anisotropy (FA), fiber count (FiberConn), node degree (Deg), cluster coefficient (ClusC) and relative standard-uptake-values (rSUV). Receiver Operating Characteristic (ROC) curves were used to evaluate and compare the diagnostic accuracy of the most significant metrics and brain regions and expressed as area under the curve (AUC). Comparisons were performed between groups. The RH-Accumbens/Deg demonstrated the highest AUC when differentiating between CTRLEMCI (82%), whether rSUV presented it in several brain regions when distinguishing CTRL-LMCI (99%). Regarding CTRL-AD, highest AUC were found with LH-STG/FiberConn and RH-FP/FiberConn (~100%). A larger number of neuroimaging metrics related with cortical atrophy with AUC>70% was found in CTRL-AD in both hemispheres, while in earlier stages, cortical metrics showed in more confined areas of the temporal region and mainly in LH, indicating an increasing of the spread of cortical atrophy that is characteristic of disease progression. In CTRL-EMCI several brain regions and neuroimaging metrics presented AUC>70% with a worst result in later stages suggesting these indicators as biomarkers for an earlier stage of MCI, although further research is necessary.

